# Improvement of Biocatalytic Properties and Cytotoxic Activity of L-Asparaginase from *Rhodospirillum rubrum* by Conjugation with Chitosan-Based Cationic Polyelectrolytes

**DOI:** 10.3390/ph15040406

**Published:** 2022-03-27

**Authors:** Natalia V. Dobryakova, Dmitry D. Zhdanov, Nikolay N. Sokolov, Svetlana S. Aleksandrova, Marina V. Pokrovskaya, Elena V. Kudryashova

**Affiliations:** 1 Chemical Faculty, Lomonosov Moscow State University, Leninskie Gory St. 1, 119991 Moscow, Russia; natdobryak@gmail.com; 2 Laboratory of Medical Biotechnology, Institute of Biomedical Chemistry, Pogodinskaya St. 10/8, 119121 Moscow, Russia; sokolov2144@yandex.ru (N.N.S.); v-aleksandrov@yandex.ru (S.S.A.); ivan1190@yandex.ru (M.V.P.); 3 Department of Biochemistry, Peoples’ Friendship University of Russia (RUDN University), Miklukho-Maklaya St. 6, 117198 Moscow, Russia

**Keywords:** L-asparaginase, *Rhodospirillum rubrum*, catalytic activity, cationic polymers, polyelectrolyte complexes, covalent conjugates, antitumor activity

## Abstract

L-asparaginases (L-ASNases, EC 3.5.1.1) are a family of enzymes that are widely used for the treatment of lymphoblastic leukemias. L-ASNase from *Rhodospirillum rubrum* (RrA) has a low molecular weight, low glutaminase activity, and low immunogenicity, making it a promising enzyme for antitumor drug development. In our work, the complex formation and covalent conjugation of the enzyme with synthetic or natural polycationic polymers was studied. Among non-covalent polyelectrolyte complexes (PEC), polyethyleneimine (PEI) yielded the highest effect on RrA, increasing its activity by 30%. The RrA-PEI complex had increased stability to trypsinolysis, with an inactivation constant decrease up to 10-fold compared to that of the native enzyme. The covalent conjugation of RrA with chitosan-PEI, chitosan-polyethylene glycol (chitosan-PEG), and chitosan-glycol resulted in an increase in the specific activity of L-asparagine (up to 30%). RrA-chitosan-PEG demonstrated dramatically (by 60%) increased cytotoxic activity for human chronic myeloma leukemia K562 cells in comparison to the native enzyme. The antiproliferative activity of RrA and its conjugates was significantly higher (up to 50%) than for that of the commercially available EcA at the same concentration. The results of this study demonstrated that RrA conjugates with polycations can become a promising strategy for antitumor drug development.

## 1. Introduction

L-Asparaginases (L-ASNases; E.C. 3.5.1.1) belong to the amidohydrolases class that hydrolyzes L-asparagine to L-aspartic acid and ammonia. Bacterial L-asparaginases are used to treat various types of leukemia, particularly acute lymphoblastic leukemia (ALL) [1,2,3]. The main therapeutic effect of L-ASNases is based on a decrease in the concentration of L-asparagine in the blood, as a result of which the synthesis of nucleic acids and proteins is disrupted, leading to the death of cancer cells. Unlike normal cells, leukemic cells lack or have reduced asparagine synthetase activity; thus, cancer cells are more dependent on L-asparagine concentrations in the microenvironment. In this regard, leukemic cells are more sensitive to L-ASNases. Other L-ASNase substrates include L-glutamine, D-asparagine, succinic acid monoamide, and asparaginyl-tRNA [4,5]. Thus, therapeutic efficacy and antiproliferative effects may also depend on their degradation. Currently, native L-ASNase from *Escherichia coli* (EcA) or *Dickeya dadantii* (formerly known as *Erwinia chrysanthemi*, ErA) along with a pegylated form of EcA are successfully used for the treatment of patients with acute lymphoblastic leukemia [6,7,8,9]. However, L-ASNases have rather strong immunogenic properties [10] and some side effects (i.e., hepato- and neurotoxicity) [11], which limits their use in clinics. In this regard, the development of L-ASNases and their biobetters with improved pharmacological properties has become an important challenge. One of the attractive enzymes is L-ASNase from *Rhodospirillum rubrum* (RrA), which has a relatively low molecular mass (172 aa; 18 kDa) and a low degree of homology to EcA or ErA [12]. Despite the relatively high Michaelis constant and lower catalytic activity for L-asparagine in comparison to EcA, this enzyme has a more pronounced cytotoxic activity on cancer cells. The phenomenon of higher-than-expected cytotoxic activity of RrA can be explained by the existence of asparaginase-independent mechanisms. For instance, RrA could suppress telomerase activity in several human cancer cell lines, normal activated CD4^+^ T lymphocytes, and xenografts of human solid tumors [13,14,15]. Moreover, there is indirect evidence that RrA can be internalized via a receptor-mediated mechanism, which may also contribute to its cytotoxic activity, which is higher than predicted based solely on asparaginase activity.

An important advantage of RrA is also that it has a lower L-glutaminase activity, which is less than 0.1% of L-asparaginase activity. The disadvantage of this asparaginase is the alkaline optimal pH of 9.0–9.3, which decreases its activity under physiological conditions. In addition, the enzyme is not stable enough in the bloodstream and is sensitive for blood proteolytic enzymes.

To improve the pharmacological characteristics of L-ASNases, conjugation with polymers is used. Covalently modified polyethylene glycol (PEG) EcA (Oncaspar) is commonly used for the treatment of leukemia and has a prolonged half-life in the bloodstream and reduced immunogenicity [16,17]. However, this modification does not always reduce the manifestation of side effects. Previously we found that conjugation with the PEG–Chitosan branched copolymer [18,19], chitopegylation, efficiently increased the catalytic activity under physiological conditions (pH 7.5) by the factor of 3–4 for L-ASNase from *Erwinia carotovora* (EwA), mainly due to the shift in the pH optimum from pH 8.8 to more acidic conditions by 1–1.5 units. Another important characteristic determining the pharmacological properties of bioconjugates is the branching of the copolymer, which affects the availability of the enzyme to plasma proteases and immune cells and has a stabilizing effect. These observations make the approach based on forming conjugates of L-ASNase with polyelectrolytes a promising strategy for improving pharmacological properties. Applying a similar graft copolymer conjugation approach to RrA might yield an enzyme with superior pharmacological properties.

## 2. Results

### 2.1. Synthesis and Characterization of Copolymers

To optimize the physicochemical parameters of RrA, the influence of some polyelectrolytes of various compositions was studied in the work on the activity of the enzyme under study (Table 1). The following polyelectrolytes were used in the work: chitosan–PEG, chitosan-glycol, chitosan-PEI, PEI, PEI-PEG, and heparin. These polymers differed both in their composition and molecular structure.

**Figure 1:**
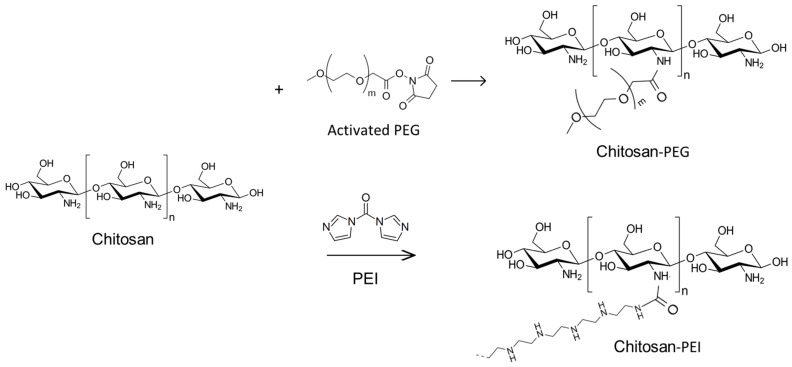
Schemes of reactions for the synthesis of chitosan copolymers with polyethylene glycol (PEG) or polyethyleneimine (PEI).

To obtain covalent conjugates of RrA, the grafted chitosan copolymers with a branched structure were synthesized. The synthesis schemes are shown in Figure 1. Chitosan copolymers with PEG were obtained by chemical modification of deacylated chitosan amino groups with an activated PEG derivative with high reactivity. To synthesize the chitosan-PEI copolymer, chitosan and PEI were conjugated by amino groups via carbonyldiimidazole.

**Figure 2:**
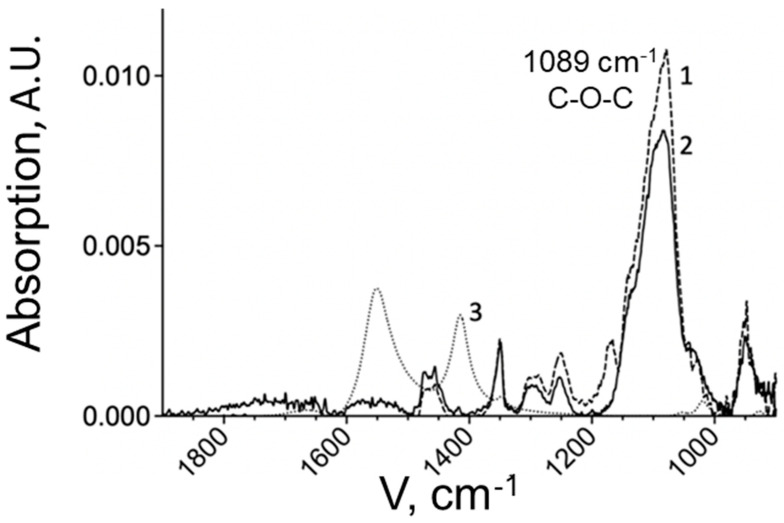
IR spectra of activated PEG 5 mg/mL (**1**), chitosan-PEG 14 chain copolymer 5 mg/mL (**2**), chitosan 5 kDa 1 mg/mL (**3**) in 10 mM PBS pH 7.3, 25 °C. A.U., arbitrary units.

The composition of the obtained chitosan copolymers was studied by FTIR spectroscopy. Figure 2 represents the IR spectrum of chitosan-PEG with 14 PEG chains in comparison with chitosan MW 5 kDa and PEG MW 5 kDa.

**Figure 3:**
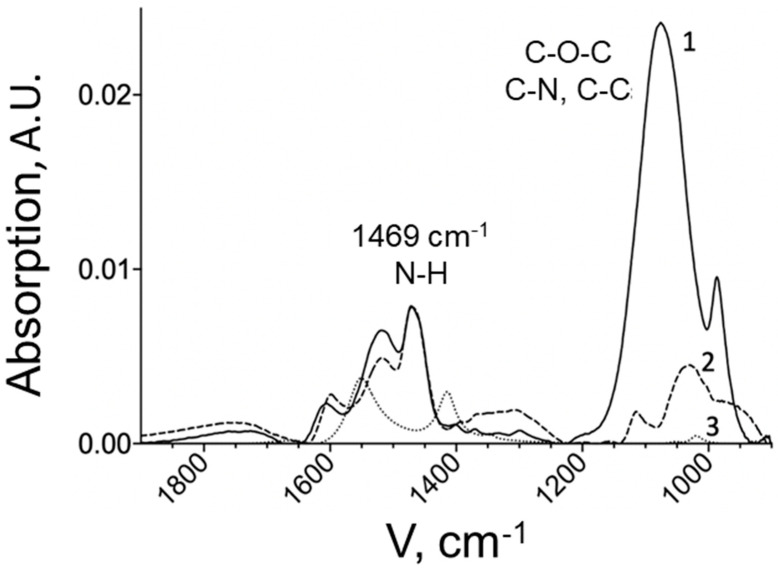
IR spectra of chitosan-PEI copolymer (**1**), PEI 10 mg/mL (**2**), chitosan 5 kDa 1 mg/mL (**3**) in 10 mM PBS pH 7.5, 25 °C. A.U., arbitrary units.

The graph shows an absorption band distinctive for chitosan in the region of 1600–1500 cm^−1^, corresponding to the fluctuations of free amino groups remaining in chitosan after modification. The degree of chitosan PEGylation was determined by the intense absorption band characteristic of the PEG derivative at 1089 cm^−1^, corresponding to fluctuations in C-O-C bonds [20]. The concentration of PEG was determined using a calibration dependence. Due to the presence of the band in the spectrum of the copolymer, it can be concluded that the modification of chitosan was successful. In the spectrum of the chitosan-PEI copolymer (Figure 3), the main peak for PEI is at 1459 cm^−1^, which is responsible for fluctuations in N-H bonds. This peak is also present in the copolymer. The quantitative content of PEI in the copolymer was determined by its intensity.

An increase and expansion of the peak in the region of 1000–1150 cm^−1^ was observed for chitosan-PEI, which indicates the formation of new C-N and C-C bonds with chitosan. This peak also includes fluctuations in the C-O-C bonds of the pyranose part of chitosan, but it is significantly less pronounced.

The composition and characteristics of all chitosan copolymers used in the work are presented in Table 2.

### 2.2. The Influence of Polyelectrolytes on RrA Activity

The variation in the surface charge of the enzyme during conjugation with polyelectrolytes can affect the binding to the substrate, adsorption on cells, and interaction between subunits in the protein, which is important in the case of the tetrameric enzyme RrA, the activity of which strongly depends on the interunit interaction. Polyelectrolytes and proteins can spontaneously and reversibly bind to each other in solution, forming protein-PEC due to the electrostatic interactions between the charged amino acid residues of the enzyme and the ionic groups of the polymer. As shown for several systems, the formation of PEC protects the enzyme from contact with the components of the medium, optimizes the surface charge, and provides optimal pH under physiological conditions [21,22].

The surface charge of the enzyme determines the binding efficacy to the polyelectrolytes. At a neutral pH, RrA will have a larger negative charge, and polycations will actively bind to the enzyme, which may stabilize its conformation. For comparison, another L-ASNase, EwA, which was intensively studied in our laboratory, was used in the experiments. For EwA and RrA, the ratio of positively and negatively charged amino acids was different, leading to the difference in isoelectric point (pI) shown in Table 3. Interaction with polyelectrolytes is likely to have different effects on the properties of these two enzymes, especially on activity.

It was found that the activity of RrA in 10 mM Tris-HCl pH 7.5 was significantly increased (up to 1.5 times) due to the presence of positively charged tertiary amino groups shielding negatively charged groups in RrA. Sodium–phosphate buffer does not affect the activity of the enzyme [25]. Most likely, polycations oligo- and polyamines due to multipoint electrostatic interactions and the formation of strong PEC with the enzyme (K_D_ ≈ 10^−6^–10^−7^ M [26]) can increase the activity of RrA. Thus, polymers such as chitosan and PEI were used in our study.

To select the polyelectrolyte responsible for the highest increase in activity, the rate of hydrolysis of L-asparagine by RrA-PEC was measured by CD spectroscopy. The following RrA conjugates were used: heparin 15–20 kDa, PEI 30–40 kDa, branched PEI-PEG 13 kDa, chitosan 7 kDa, chitosan-PEG, and chitosan-glycol 72 kDa.

**Figure 4:**
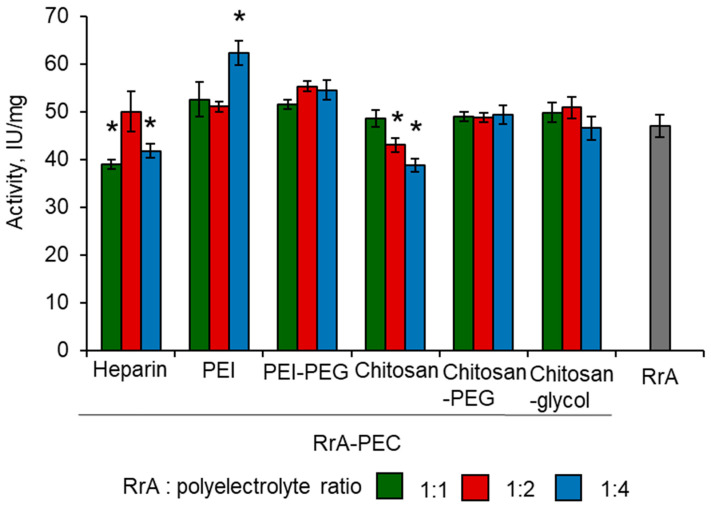
The enzymatic activity of RrA-PEC to 20 mM L-asparagine in 10 mM PBS pH 7.5. PEC, polyelectrolyte complex; PEG, polyethylene glycol; PEI, polyethyleneimine; RrA, *Rhodospirillum rubrum* L-ASNase. N = 4. * *p* ≤ 0.05 relative to the activity of non-complexed RrA.

Figure 4 represents the results of measuring the activity of RrA-PEC. RrA-PEI demonstrated an increase in activity in a ratio of protein–polyelectrolyte of 1:4 by 30% in comparison to the native enzyme. A similar effect can be observed for the branched copolymer PEI-PEG. For complexes with chitosan and heparin, the opposite effect was observed: an increase in polyelectrolyte resulted in a decrease in activity by approximately 10–20%. Chitosan-PEG and chitosan-glycol resulted in a slight and insignificant increase in the activity of RrA.

Polyelectrolytes can influence the rate of binding of the substrate to the enzyme, which can be estimated using the Michaelis constant. K_M_ is a key parameter since it can be used to determine substrate concentrations at which the active center of the enzyme is saturated. The concentration of L-asparagine in the bloodstream is small (less than 1 mM), and the enzyme at relatively high values of K_M_ will have activity below the maximum. Therefore, it is important to determine the effect of polyelectrolytes on the binding of RrA to the substrate.

The most active PEC–RrA-PEI was studied in comparison with the initial enzyme. To identify binding affinity, K_M_ values were determined at pH 6.5, 7.5, and 9.0 for the native enzyme and RrA-PEI (Table 4).

The results demonstrated that the formation of PEC with PEI has a negligible effect on K_M_. When reaching pH 9.0, K_M_ decreased slightly for both the native RrA and RrA-PEI. As expected, the activity at the optimum pH of 9.0 is higher for both the native enzyme and the PEC compared to the activity level at pH 6.5 and 7.5. The V_max_ value for PEC (RrA-PEI) was higher than that for native RrA over the entire studied range of pH values. Thus, this polyelectrolyte does not impair binding to the substrate and increases the rate of hydrolysis of L-asparagine; therefore, it may be promising for further optimization of the properties of the enzyme.

More pronounced effects are expected for covalent conjugates. As mentioned earlier, conjugates allow the creation of strong bonds of the polymer with the enzyme and obtain a more stable drug. The use of these conjugates for intravenous administration as a drug is likely to be more effective.

### 2.3. Preparation and Properties of Covalent Conjugates

To study and optimize the properties of RrA, conjugates with chitosan copolymers of various compositions were synthesized. The copolymers used are shown in Table 2 above. RrA conjugates were obtained by reductive amination through the Schiff base formation reaction by protein amino groups. As a result, so-called “crown-shaped” conjugates were obtained.

Chitosan with molecular weights of 5 and 7 kDa and different rates of PEGylation (14–23 PEG chains) was used for the synthesis of conjugates. The concentration of RrA and the number of copolymer chains in the produced conjugates were determined by UV and IR spectroscopy. The number of copolymer chains per enzyme molecule was determined according to the concentration of the enzyme and the copolymer. 

**Figure 5:**
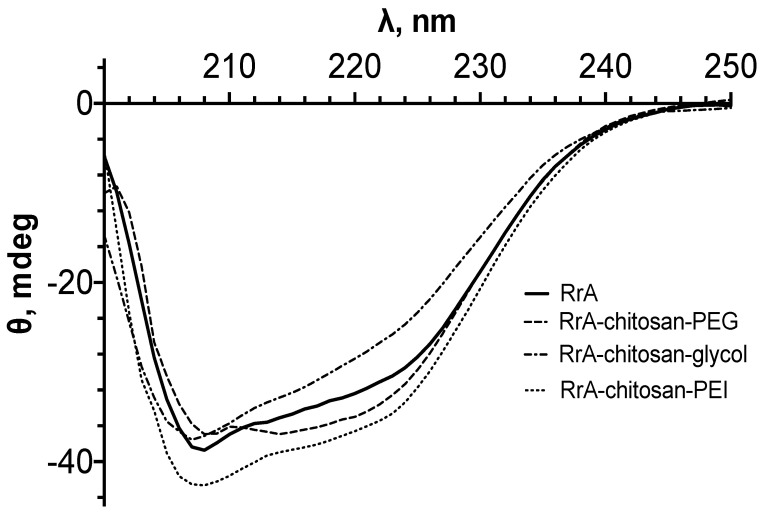
CD spectra of native RrA and its conjugates RrA-chitosan-PEG, RrA-chitosan-glycol and RrA-chitosan-PEI. C(RrA) = 1 mg/mL, 0 mM sodium phosphate buffer, 37 °C, pH 7.5.

The content of chitosan-PEG (or other copolymer studied) in the RrA conjugate samples was determined using the known PEGylation degree of chitosan (or modification degree of chitosan by PEI) from the intensity of the characteristic PEG (or glycol or PEI) absorption band at 1089–1100 cm^−1^ in the IR spectrum, according to our previously developed method [20]. The absorbance of the enzyme was determined from the amide I peak in the IR spectrum for the conjugate. The example of the RrA conjugate spectra is presented in Figure S1 (in Supplementary Materials). The number of copolymer chains per protein globule was determined using the calibration curves of the dependence of the amide I absorbance band’s intensity on the enzyme concentration (Figure S2). The concentration of the enzyme in the conjugates was also controlled by CD spectroscopy (Figure 5). The resulting composition and specific activities of conjugates are shown in Table 5.

CD spectrometry was also used to characterize the secondary structure of the synthesized RrA conjugates in comparison with the native enzyme. Figure 5 shows the CD spectra of RrA-chitosan-PEG, RrA-chitosan-glycol, and RrA-Chitosan-PEI conjugates. The spectra of the conjugates do not differ from those of the native enzyme. As can be seen in Figure 5, the spectra of RrA and its conjugates with chitosan copolymers are similar in shape. Both spectra contain the negative maxima at the wavelengths of 210 and 222 nm. Such a pattern corresponds to the structure of a protein with a high content of α-helices. 

The content of the secondary structure elements in the conjugates was calculated from the CD spectra. The parameters of the secondary structure of the conjugates from the CD spectroscopy data determined using the CDNN program are presented in Table 6. Quantitative analysis of the data obtained using the CD spectroscopy demonstrated that the content of the secondary structure elements in RrA changed only slightly on the formation of a conjugate. 

The zeta potentials of the native enzyme and conjugates were measured. Table 5 shows that RrA in a neutral solution has a negative zeta potential. On the contrary, RrA-chitosan-PEG and RrA-chitosan-glycol conjugates have an increased zeta potential due to modification by polycations.

The catalytic activity was determined by CD spectroscopy as described above. For all conjugates, the enzymatic activity increased in comparison to the native enzyme. The highest increase in specific activity (up to 32%) was observed for the conjugate RrA-chitosan-PEG 7_23 (chitosan 7 kDa, 23 chains of PEG, Table 5). For a conjugate with chitosan of 5 MW kDa and a lower PEGylation degree, the activity increased by only 10%. The degree of PEGylation can also affect the catalytic activity of L-ASNase, as shown for EwA [27]. With an increase in PEG chains from 14 to 23 in RrA conjugates, the activity of the enzyme also increased. For the RrA-chitosan-glycol conjugate, the effect was not so significant and practically did not differ from the PEC. The activity of the RrA-chitosan-PEI conjugate (55–57 IU/mg) was higher than that of the PEC complex with this copolymer (51–53 IU/mg) (Figure 4). Thus, covalent modification makes it possible to achieve a stronger effect in the activity regulation of RrA.

### 2.4. The Dependence of the Activity of the Native Enzyme and Conjugates on pH

**Figure 6:**
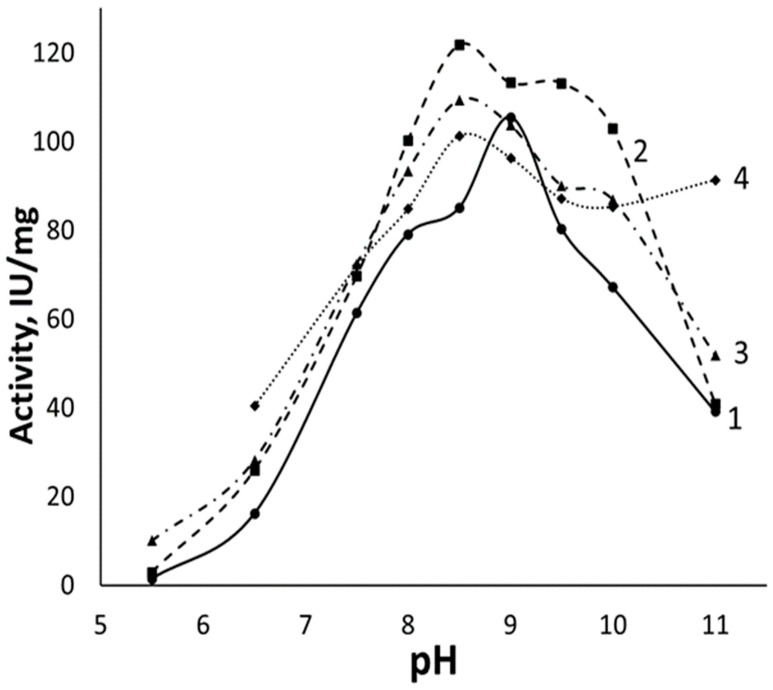
pH dependence of the specific activity of (**1**) native RrA, (**2**) RrA-chitosan-PEG 7_23, (**3**) RrA-chitosan-glycol, and (**4**) RrA-chitosan-PEI in a 5 mM citrate–phosphate–borate buffer at 37 °C.

As shown above, RrA conjugation with polycations increases the activity of L-ASNase by up to 32%. One of the reasons for this effect may be a shift in the pH of the optimum activity for RrA-PEC toward physiological values (pH 7.5). To test this hypothesis, the activity of native RrA and its conjugates with chitosan copolymers was determined in the pH range of 5.5–11.0 (Figure 6).

RrA was most active at pH 9.0, which makes it similar to EwA, having a pH optimum in the alkaline media at pH 8.5–8.8. Native RrA lost approximately 40% of its maximum activity in a solution with a physiological pH (at pH 7.5).

RrA conjugates chitosan-PEG 7_23 (chitosan 7 kDa, 23 chains of PEG), chitosan-glycol, and chitosan-PEI show a broadening of the optimal pH interval, as well as a shift in maximum activity by 0.5 units into a more acidic condition compared to native RrA (Figure 6). This may be due to the polycationic properties of chitosan and PEI. They change the local pH near the surface of the enzyme and the active center, making the pH different from that in the solution.

### 2.5. The Stability of RrA Conjugates to Trypsinolysis

**Figure 7:**
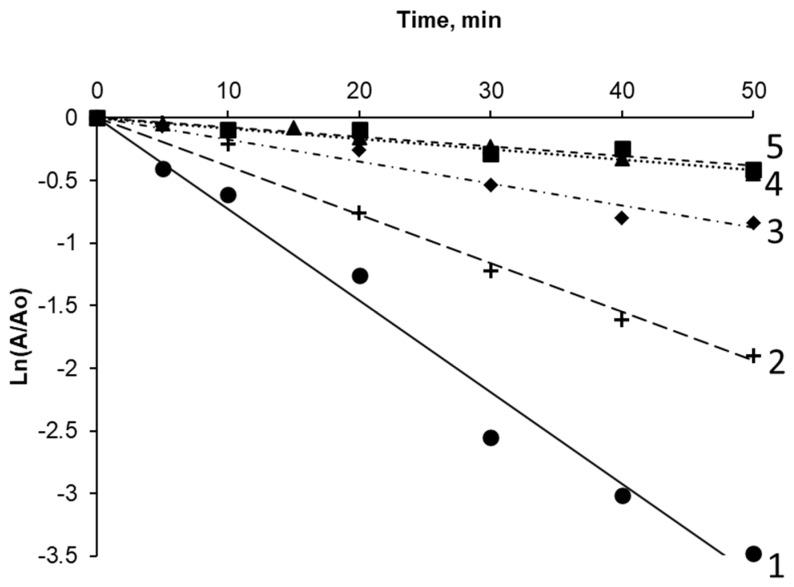
Semilogarithmic dependence of hydrolysis activity of 20 mM L-asparagine after incubation with 0.0025 mg/mL trypsin at 37 °C for 1 mg/mL native RrA (**1**) and conjugates: RrA-chitosan-glycol (**2**), RrA-chitosan-PEG 7_23 (**3**), RrA-chitosan-PEI (**4**), and RrA-PEI (**5**).

Resistance to trypsinolysis correlates with the availability of the enzyme surface to blood plasma factors, including proteins of the complement system. In addition, trypsin-like proteases present in the bloodstream can lead to the degradation of the enzyme and its rapid withdrawal from the body, which significantly reduces the effectiveness of the drug. The curves of enzyme inactivation during conjugate trypsinolysis in comparison with the native enzyme are shown in Figure 7.

The interaction of the enzyme with polyelectrolytes, both covalent and non-covalent, led to a significant increase in the stability of the enzyme to trypsinolysis. Table 7 represents the values of the inactivation constants. The inactivation constants for conjugates with chitosan-PEG 7_23 and chitosan-PEI decreased by 4 and 9 times, respectively. For RrA-PEI, the inactivation constant was 10 times lower than that for native RrA.

### 2.6. Cytotoxic Activity of RrA Conjugates

For the most promising catalytically active and stable RrA conjugates, the antiproliferative activity was studied in comparison with commercial EcA (“Vero-asparaginase”) and EwA. The experiment was performed on chronic myeloid leukemia K562 cells, which have relatively high asparagine synthetase activity. With a lack of L-asparagine, the cells can synthesize this non-essential amino acid, which reduces their sensitivity to the action of the enzymes. At the same time, they retain the sensitivity to RrA. Moreover, the antiproliferative activity on these cells is higher for RrA than for EwA, regardless of its lower L-asparaginase activity [12]. This suggests that the cleavage of L-asparagine is not the only mechanism for the manifestation of the cytostatic activity of asparaginases. Thus, the possibility of internalization of the enzyme into cells through interaction with specific receptors of tumor cells is one of the factors affecting the antiproliferative activity of L-ASNase. In particular, it has been shown that in addition to the ability to hydrolyze L-asparagine, RrA can internalize into leukemic cells and suppress telomerase activity, which affects the antitumor activity of the enzyme [15,28]. Other studied L-asparaginases are not able to penetrate the studied cells. At the same time, there are L-ASNases superior in their cytostatic activity to RrA. Thus, no direct correlations between the properties of the enzyme and its cytotoxic activity have been found, and the question of the specific mechanisms of action of L-ASNase preparations from various sources requires additional studies.

**Figure 8:**
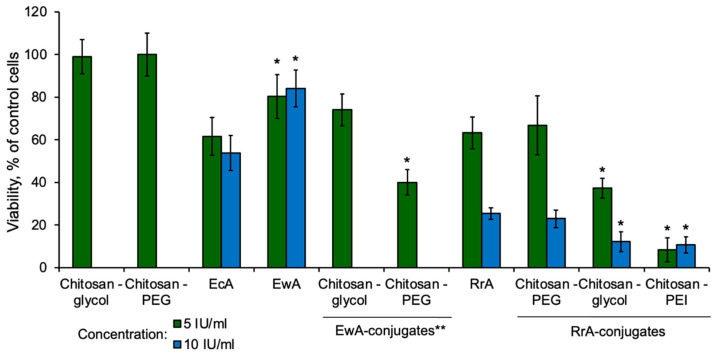
Cytotoxic activity of EwA, EcA, RrA, and EwA and RrA conjugates toward the K562 cell line. EcA, *Escherichia coli* L-ASNase; EwA, *Erwinia carotovora* L-ASNase; covalent conjugates EwA and RrA; PEG, polyethylene glycol; PEI, polyethyleneimine; RrA, *Rhodospirillum rubrum*. N = 4. * *p* ≤ 0.05 vs. cells treated with non-conjugated RrA. ** Data obtained earlier and published in the article [19].

Figure 8 represents the cytotoxic activity of conjugates RrA-chitosan-PEG 5_14, RrA-chitosan-glycol, and RrA-chitosan-PEI. The antiproliferative activity of RrA and its conjugates was significantly higher than that of the commercially available drugs EcA at the same concentration. The most significant decrease in cell survival was demonstrated for RrA-chitosan-glycol (12.2 ± 4.7%) and RrA-chitosan-PEI (10.6 ± 3.7%) conjugates at a concentration of 10 IU/mL. For EwA and EcA, the percentage of surviving cells was not less than 53.4 ± 8.2% and 84 ± 8.7%, respectively. It should be mentioned that free chitosan copolymers did not show cytostatic activity. The absence of cytotoxic activity of chitosan and its copolymers against K562, Jurkat, and Raji cell lines was also demonstrated in our previous work when we studied L-asparaginase conjugates from *Erwinia carotovora* [19].

## 3. Discussion

Modification of chitosan with PEG makes it possible to obtain a copolymer with a different number of free amino groups, which affects the overall positive charge of the resulting copolymer in solution, as well as the charge distribution on the surface of the enzyme during conjugation. By varying the degree of chitosan pegylation, it is possible to choose the optimal copolymer for conjugation and to increase the activity and cytotoxicity of drugs against leukemic cells, as has been shown for EwA [19,27]. For the same purposes, pegylated PEI was used in the work. PEG reduces the toxicity of the polycation by neutralizing and screening the primary amino groups. Chitosan was used to optimize the charge of linear PEI as it can increase the biocompatibility of the polymer. The chitosan-PEI copolymer is used for safe gene delivery techniques [29,30]. Heparin, as well as the less sulfated polysaccharides glucosaminoglycan and heparan sulfate, can effectively bind to proteoglycans on the cell surface and extracellular matrix. Such priorities provide better protein adsorption on cells and longer circulation in the bloodstream. Moreover, binding to heparan sulfate reduces susceptibility to proteolytic degradation [31].

The zeta potentials of chitosan solutions at neutral pH are approximately 15–20 mV [32], and PEI is approximately 30–40 mV [33], depending on the molecular weight. For the control, heparin (polyanion) containing negatively charged sulfuric groups (the degree of sulfation is more than 80%) and having a zeta potential of −25–−35 mV [34] was used.

Due to the presence of several primary and secondary amino groups, PEI and PEI-PEG have a positive charge in a solution with a neutral pH value and can effectively bind to negatively charged amino acid residues of RrA, forming PEC. Polycations change the microenvironment of the active center, stabilize the conformation of the protein, and can improve the catalytic parameters. The result of such modifications is a higher activity of the enzyme in non-optimal conditions. However, unlike Tris buffer, which can increase the activity of RrA in millimolar concentrations (10 mM) and under physiological conditions (pH 7.5, 37 °C), enzyme–polyelectrolyte complexes are much more stable due to the presence of multiple electrostatic interactions and are, therefore, promising for further use. Non-modified chitosan and heparin, probably due to their more rigid structure, reduce the mobility of the protein globule, which leads to a slight deterioration in the catalytic functions of the enzyme. Even though heparin reduces the activity of L-ASNase, this polyelectrolyte can increase the stability of the enzyme preparation and its sorption on cells after intravenous administration. The pegylation of chitosan amino groups contributes to an increase in the segmental mobility of the polysaccharide, reduces the concentration of positive charges, and achieves a milder effect on the enzyme. In addition, PEG itself can reduce the toxicity of L-ASNase preparations, allergic reactions, and other side effects during treatment.

For comparison, other effects with these polyelectrolytes were previously observed for the enzyme EwA. For example, the activity of EwA-PEC with chitosan-glycol was found to be almost two times lower than that of the native enzyme [19]. When PEC was formed with PEI and chitosan (90 kDa), the activity of EwA also decreased.

**Figure 9:**
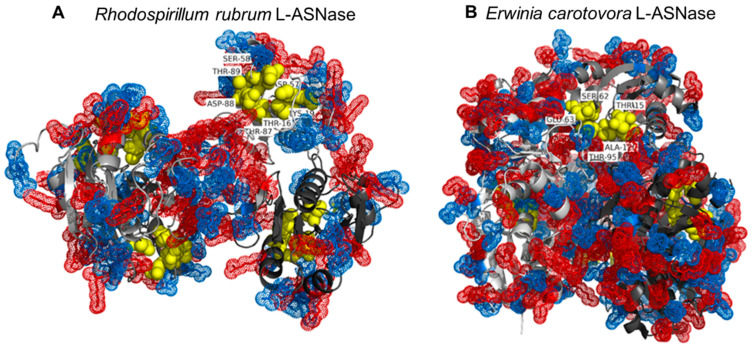
Three-dimensional model of tetrameric structures of (**A**) RrA and (**B**) EwA. Asp and Glu residues are highlighted in blue, Arg and Lys are highlighted in red, and amino acid residues belonging to the active center of L-asparaginases are highlighted in yellow (RrA: Thr16, Lys19, Asp57, Ser58, Thr87, Asp88, Thr89; EwA: Thr15, Ser62, Glu63, Thr95, Asp96, Ala120). Four subunits are colored gray.

Since RrA and EwA have different structures and catalytic properties, polyelectrolytes will have different effects on their activity. To understand the possible reason for such a difference, 3D structures of these enzymes were obtained using the PyMOL program (Figure 9).

The presence of sites with positively charged (arginine and lysine) and negatively charged (aspartic and glutamic acids) amino acids over the entire surface determines the binding capacity of enzymes to both polycations and polyanions. Multiple negative charges, perhaps, allow RrA to bind polycations more effectively. However, as seen from the activity results, heparin also affects the activity of the enzyme and, therefore, interacts with the protein globule.

In the case of EwA, negatively charged amino acid residues predominate on the protein surface. The EwA model also demonstrates tighter contact between the subunits at the junction of which the active center is located. It is possible that polycations, breaking the interunit bonds, have a stronger effect on catalysis. In addition, the quaternary structure in this enzyme is mainly supported by interactions between arginine, glutamate, and aspartate residues [35], and a high concentration of amino groups can disrupt the ionic bonds between these residues and destabilize the enzyme. Thus, the amino groups of polycations have the opposite effect on EwA, destabilizing the enzyme and reducing its activity.

Conjugation of RrA with polycations increased the activity of the enzyme in a more acidic environment. Previously, for EwA, we have also observed the shift in the pH optimum to a more acidic condition and an increase in activity by 3–4 times due to chitopegylation [23,27]. Modification with pegylated chitosans having MW 3–160 kDa demonstrated that an increase in activity is not only associated with a shift in the optimum pH. Polyelectrolytes can also stabilize interactions between enzyme subunits, affecting the equilibrium between dimeric and tetrameric forms. Conjugates with 7–15 kDa chitosan turned out to be the most optimal, allowing increasing activity at pH 7.5 without disturbing the protein conformation. In our previous studies, it was found that the zeta potential was approximately 0 mV at pH 7.5 for native EwA. Conjugates whose zeta potentials did not exceed 1–3 mV were the most active. Pegylated chitosan with an MW of 7–15 kDa is among such conjugates. PEG chains shield part of the positive charges of chitosan amino groups, preventing the shift of the surface charge of the enzyme to the suboptimal region. For conjugates with non-pegylated chitosans, as well as with chitosan-glycol, the zeta potential was several dozen mV, leading to a decrease in the activity by several times. The increased positive charge of the conjugate surface can affect the catalysis due to the difficult release of the hydrolysis product, L-aspartic acid, from the active center. The opposite situation was observed for RrA. Due to the predominantly negative surface charge of the protein, conjugation with polycations having a higher charge, as in glycol-chitosan and PEI, can probably be optimal. Indeed, for RrA conjugates with chitosan-glycol and chitosan-PEI, the activity increased significantly.

Chitosan copolymers also improved resistance of RrA to trypsin hydrolysis. A similar effect was previously observed for low molecular weight pegylated chitosan (3 kDa), which allowed an increase in the resistance to trypsinolysis by two times for EwA. The increase in the stability of the native enzyme when using high molecular weight glycol-chitosan was almost at the same level (1.6 times) as for chitosan-PEG [27].

The resistance of conjugates to the action of trypsin may be due to several factors. Due to the presence of a number of positive charges, such as in the main polyelectrolytes of PEI and chitosan-PEI, better binding of copolymers to the protein globule and more effective shielding of the enzyme from contact with trypsin is provided. The degree of branching may play a role. The branched copolymers chitosan-PEG and chitosan-PEI are probably better at blocking the positive groups of lysines and arginines on the enzyme surface from binding to trypsin in comparison to linear chitosan-glycol. There is also a possibility that the stabilization of the conformation of the enzyme affects the resistance to trypsinolysis [36]. Copolymers can increase the overall stability of the protein globule by fixing the conformation and limiting the segmental mobility of L-ASNase. Thus, the unfolding of the protein is prevented during primary cleavage by the protease, and the enzyme remains active. In addition, with partial trypsinolysis, the aggregation of the enzyme can occur as a result of the unfolding of the protein, and polyelectrolytes protect the enzyme from aggregation due to the electrostatic repulsion of similarly charged particles.

Thus, the tendency of the influence of chitosan copolymers persists for L-ASNases from different sources, and it can be assumed that resistance to trypsinolysis depends more on the nature of the polyelectrolyte, i.e., branching and the amount of lysine and arginine residues available for protease on the protein surface, rather than on the surface charge of the enzyme.

According to our previous results, the EwA conjugate with chitosan-PEG most actively (up to 40%) suppressed cell growth at a dose of 5 IU/mL, which was more than two times more effective than the native enzyme [19]. For RrA, no such strong effect was observed in the case of pegylated chitosan. For EwA conjugates with chitosan-glycol, the cytotoxicity was also 10–20% higher than that of the native enzyme. It was also shown that the studied chitosan-based copolymers by themselves do not have a significant effect on cell survival [19].

Thus, for enzymes modified by copolymers, a more pronounced antiproliferative effect was observed on K562 leukemia cells. Cytotoxicity may depend on surface charge, which is different for native enzymes and their conjugates. The enzyme preparation RrA-chitosan-PEI, due to the positive charge of the copolymer, can be better adsorbed on the surface of cancer cells, whose zeta potential is lower than that in healthy cells and varies from approximately −10 to −40 mV for different cell types [37]. Thus, the enzyme can concentrate on the membrane of tumor cells, which leads to a decrease in the content of L-asparagine in its vicinity. The amino acid deficiency created in this way provides effective antitumor activity. The same concerns affect the conjugate with chitosan-glycol, which has an increased zeta potential due to the presence of free amino groups. The zeta potential of this conjugate is approximately 0 mV, while that of the native enzyme is approximately −9 mV. Thus, we can assume that neutralization of the negative charge of the enzyme increases its adsorption on cells and cytotoxicity. For RrA-chitosan-PEG, the surface charge of the particles slightly increased, but no significant effect on tumor cells was observed. As a result, it can be expected that positively charged polyelectrolytes will provide better adsorption of conjugates on tumor cells. Perhaps this can also enhance the internalization of the enzyme inside the cells. In addition to the mentioned effects, copolymers provide stabilization of the enzyme and protect it from dissociation and degradation in the nutrient medium.

## 4. Materials and Methods

### 4.1. Preparation of Protein–Polyelectrolyte Complexes (PECs)

The enzymes RrA and EwA were previously obtained in the laboratory of medical biotechnology at the Institute of Biomedical Chemistry [25,38]. The RrA gene was isolated from the *Rhodospirillum rubrum* strain (collection of the Microbiology Department, Lomonosov Moscow State University) using the pET23a vector (Novagen) and the following primers: GCCCCTTCCCTTGCCACAGG, GGACACCCAAGCTTCCCTTTTCCG, CACAGGATCCTCAAGGCAAATGGCCG. The active producer was cultivated in Erlenmeyer flasks (the 1 L size) in 200 mL of LB medium with ampicillin (100 μg/mL) using a GFL 3033 shaking incubator (Germany) at 37 °C. The cell culture density was determined using an Aquarius 7000 spectrophotometer at 600 nm and expressed in optical units (OD600). The inducer (lactose, IPTG) was added to the medium at OD600 of 0.9–1.9 up to the final concentration of 0.2% and 0.001 M, respectively. After, the induction biomass was produced for 17–20 h. At the end of the incubation, cells were sedimented by centrifugation (15 min, 2500× *g*). The biomass was resuspended in buffer A (10 mM NaH2PO4, 1 mM glycine, 1 mM EDTA, pH 7.5) and sonicated in a UZDN2T disintegrator (Russia) for 10 min (1 min sonication with intervals for 1 min). The cell extract obtained by centrifugation of the sonicated suspension (60 min, 35,000× *g*) was applied onto a QSepharose column (2.0 × 30.0 cm) equilibrated with buffer A. Fractions containing RrA were diluted 10 times with buffer A pooled and applied onto a DEAE-Toyopearl 650m column (1.5 × 20.0 cm). In both cases, protein was eluted using a linear gradient of NaCl concentration (0.0–1.0 M) at the elution rates of 78 and 30 mL/h, respectively. At the final stage, the enzyme solution was desalinated and concentrated using an Amicon cell containing a Millipore filter (NMWL 18,000). All purification procedures were performed at 4 °C.

L-ASNase-PEC conjugates were obtained by mixing the enzyme at a concentration of 1.5 mg/mL and an ionogenic polymer at a ratio from 1:1 to 1:4 by mass. The solutions were incubated at room temperature for 30 min.

The copolymer of chitosan with activated PEG (chitosan-PEG) was synthesized according to a previously described protocol [27,39]. Chitosan 7 kDa (provided by the Federal Research Center “Fundamentals of Biotechnology” of the Russian Academy of Sciences) was dissolved in a 3% solution of CH_3_COOH (Paneco, Moscow, Russia) to a final concentration of 5 mg/mL. Then, the pH of the solution was adjusted to 6.5 using 10 mM phosphate-buffered saline PBS pH = 7.5 (Paneco, Moscow, Russia), and a solution of activated PEG (mPEG-suc-NHS) (Sigma-Aldrich, St. Louis, MO, USA) in DMSO (5 mg/mL) was added dropwise under stirring. PEG was taken in 2–5-fold molar excess relative to chitosan amino groups. During the reaction (2–2.5 h), the pH was maintained in the range of 7.0–7.5 (with 150 mM sodium phosphate buffer, pH 7.5). The resulting product was dialyzed using an MWCO Serva 14 kDa dialysis membrane in 10 mM PBS pH 7.5 to remove unconjugated compounds. Commercially available EcA (Veropharm) and recombinant L-asparaginase (EwA) were used as control preparations.

The copolymer of chitosan with polyethyleneimine (chitosan-PEI) 30–40 kDa was synthesized by crosslinking chitosan and PEI amino groups via 1,1′-carbonyldiimidazole (KDI) (Sigma-Aldrich, St. Louis, MO, USA) as described earlier [29]. A total of 7.5 mg of chitosan 7 kDa was dissolved in 0.5% CH_3_COOH, and the pH was adjusted to 7.0 (with 150 mM sodium phosphate buffer, pH 7.5). KDI was added to the obtained solution in a molar ratio of KDI–chitosan amino groups of 2:1, followed by constant stirring at room temperature for 1.5 h. A PEI 30–40 kDa (Serva, Heidelberg, Germany) solution at pH 7.5 was added to the chitosan-KDI solution in a molar ratio of PEI–chitosan amino groups of 1:1. The reaction was performed at room temperature overnight. The resulting copolymer was purified by dialysis against a 10 mM PBS buffer pH 7.5 using an MWCO Serva 14 kDa dialysis membrane.

The synthesis of conjugates of L-ASNase with copolymers chitosan-glycol 72 kDa (Sigma-Aldrich, St. Louis, MO, USA), chitosan-PEG, and chitosan-PEI were prepared by reductive amination with amino groups of the enzyme [23]. RrA at a concentration of 4 mg/mL in 10 mM PBS pH 7.5 was added to the above-mentioned polymers in a molar ratio of enzyme–polymer from 1:3 to 1:5. The resulting mixtures were incubated with constant stirring for 40 min at 16 °C. Then, a fivefold excess of sodium cyanoborohydride (Sigma-Aldrich, St. Louis, MO, USA) was added to the solutions and incubated for another 40 min. To purify the obtained products from the free copolymer, diafiltration using 100 kDa Amicon filters (Merch-Millipore, Kenilworth, NJ, USA) was performed in 10 mM PBS pH 7.5. The purity of the preparation was controlled by HPLC gel filtration in a Knauer chromatography system (Knauer, Berlin, Germany) on BioFox 17 SEC in a 15 cm × 1 cm^2^ column. The eluent was 15 mM PBS, pH 7.5, 150 mM NaCl; the elution rate was 0.5 mL/min, 25 °C. The chromatogram of the resulting conjugate is shown as an example of RrA-chitosan-PEG in supplemental materials (Figure S3). The resulting conjugates were lyophilized or frozen and stored at −20 °C.

### 4.2. Fourier Transform Infrared Spectroscopy (FTIR)

The FTIR spectra of RrA and its conjugates were recorded using a Tensor 27 IR Fourier spectrometer (Bruker, Ettlingen, Germany) equipped with an MCT detector cooled with liquid nitrogen and a thermostat (Huber, Offenburg, Germany). The measurements were carried out in a BioATR II thermostated cell (Bruker, Ettlingen, Germany) using a single reflection ZnSe element at 22 °C and continuous purging of the system with dry air using a compressor JUN-AIR (Gast Inc., Benton Harbor, MI, USA). An aliquot (40 µL) of the corresponding enzyme solution (0.5–1.5 mg/mL in 10 mM sodium phosphate buffer) was applied to the internal reflection element, the spectrum was recorded three times in the range from 4000 to 950 cm^−1^ with a resolution of 1 cm^−1^; performed 70-fold scanning and averaging. The background was registered in the same way and was automatically subtracted by the program. The resulting spectra were smoothed by the Savitzky–Golay method [40] to a spectral resolution of 2 cm^−1^. The spectra were analyzed using the Opus 7.0 software (Bruker, Ettlingen, Germany). 

### 4.3. Determination of the Catalytic Parameters of L-ASNase Preparations

The catalytic activity of native L-ASNase and its covalent conjugates and polyelectrolyte complexes was measured by circular dichroism (CD) using a J-815 CD spectrometer (Jasco, Tokyo, Japan) according to a previously described technique [27]. This method relies on the difference in ellipticity for optically active amino acids, including L-asparagine and L-aspartic acid, which makes it possible to observe the change in ellipticity over the time of substrate hydrolysis by L-ASNase [41]. L-asparagine (BioChemica, Billingham, UK) at a concentration of 20 mM was mixed with L-ASNase or its conjugate at concentrations of 0.03–0.035 mg/mL in 10 mM PBS pH 7.5. The reaction was performed in a quartz cuvette with a volume of 300 µL and an optical length of 1 mm in a thermostatically controlled cell at 37 °C.

To determine the pH dependence, the enzyme or its formulations at a concentration of 1 mg/mL were incubated with 20 mM L-asparagine dissolved in 5 mM citrate–phosphate–borate buffer within a pH range of 5.5–11. Ten microliters of RrA or its conjugates were added to 300 µL of the substrate, followed by CD measurement at 37 °C.

To determine the resistance to trypsinolysis, 500 µL of native enzyme or its conjugates at a concentration of 1 mg/mL was mixed with 50 µL of trypsin (Sigma-Aldrich, St. Louis, MO, USA) at a concentration of 0.0025 mg/mL and incubated for up to 60 min at 37 °C. Ten microliters of the reaction mixture was aliquoted every 5–10 min and incubated with 250 µL of 20 mM L-asparagine followed by CD measurement.

### 4.4. Determination of Zeta Potentials of L-ASNase Preparations

Determination of zeta potentials of L-asparaginase preparations was performed using “Zetasizer Nano ZS” instrument (Malvern Panalytical, Malvern, UK) in Milli-Q purified water-based solutions containing 1 mg/mL protein. The measurements were performed in a polypropylene cuvette at room temperature. To obtain each zeta potential value, 10 to 15 scans were performed and the data were averaged.

### 4.5. Molecular Modeling

Three-dimensional structures of RrA and EwA enzymes were obtained in PyMOL version 2.5.2 (Schrödinger, Inc., New York, NY, USA) software. The RrA sequence (Uni Prodcode Q2RMX1) was visualized using the SWISS-MODEL web server (http://swissmodel.expasy.org/, accessed on 23 December 2021). The EwA structure (PDB 2JK0 [42] (accessed on 23 December 2021) was downloaded from the Protein Data Bank [43] and used for complex modelling.

### 4.6. Cell Cultivation and Toxicity Assay

Human chronic myelogenous leukemia K562 cell lines (ATCC, Manassas, VA, USA) were grown in RPMI-1640 medium (Gibco, Thermo Fisher Scientific Inc., Waltham, MA, USA) supplemented with 5% fetal bovine serum (Capricorn Scientific, Ebsdorfergrund, Germany) and 1% sodium pyruvate (Paneco, Moscow, Russia) at 5% CO_2_/95% air in a humidified atmosphere at 37 °C. Cell lines were tested for mycoplasma contamination before the experiment using the Mycoplasma Detection Kit PlasmoTest™ (InvivoGen, San Diego, CA, USA).

To test acute toxicity, cells were cultivated for 72 h in a V-bottom 96-well plate (TPP, Trasadingen, Switzerland) in the presence of RrA, its conjugates or commercially available EcA (Vero-asparaginase, Veropharm, Moscow, Russia) or EwA within the range of concentrations 0–10 U/mL, and cell viability was tested by measuring the conversion of the tetrazolium salt, 3-(4,5-dimethyl-thiazol-2-yl)-2,5-diphenyltetrazolium bromide (Serva, Heidelberg, Germany), to formazan (MTT test) [44,45].

### 4.7. Statistics

Statistical analysis was performed with 2-way ANOVA and Student’s *t*-test using SPSS 25 software (IBM SPSS Statistics, Armonk, NY, USA). The results are expressed as the mean ± standard error of the mean (SEM). *p* ≤ 0.05 was considered significant.

## 5. Conclusions

In conclusion, the influence of various chitosan copolymers on the catalytic properties of RrA has been studied. The polycationic polymer polyethyleneimine and its copolymer with chitosan have the strongest effect on the properties of RrA. The cytotoxic activity of native RrA and its conjugates was higher than that of commercially available drugs (EcA), which confirms the need for further research of this enzyme and the production of new covalent conjugates to optimize their properties.

Interestingly, the cytotoxic effect did not correlate directly with asparaginase activity: the conjugates show several times higher cytotoxic activity compared to the native enzyme in the same amount of IU. Earlier, this effect was explained by receptor-mediated internalization of RrA, as supported by fluorescence microscopy and by flow cytometry. However, for L-asparaginases from other sources (EcA and WsA), for which marked cytotoxic activity on tumor cells is observed, receptor-mediated internalization was not observed [15]. Thus, again, the cytotoxic effect did not correlate directly with the impact of the receptor-mediated internalization of asparaginase. Moreover, given the fact that conjugation with branched polymers should sterically restrict RrA’s interaction with its hypothetic receptor, the internalization concept looks doubtful. Most likely, the change in zeta potential of the conjugate increases its non-specific adsorption on the cell surface, thus depleting the local concentration of asparagine near the cell. As opposed to a non-modified enzyme influencing the bulk concentration. There are multiple examples showing that zeta potential may correlate with the protein’s ability to bind to the cell surface non-specifically. Indeed, we previously tested the sorption ability of asparaginase EwA and its conjugates with PEG-chitosans on tumor cells. It was found that the conjugates of EwA enzymes with PEG-chitosans were more adsorbed on cells than the native enzyme. Adsorption for the EwA-PEG-chit conjugate was increased by 22% compared with the native enzyme. The obtained data correlated with cytostatic activity: the cytotoxicity of the EwA-PEG-chit conjugate was two-fold higher than that of the native EwA. Thus, chitoPEGylation leads to an increase in the adsorption capacity of L-asparaginase. It is probable that in the case of the RrA we studied, the same mechanism operates, due to which the cytostatic activity of the enzyme is enhanced upon conjugation. 

Whether or not this is true for RrA remains to be studied. Adsorption to the cell surface due to altered zeta is most probably a substantial factor, influencing the therapeutic enzyme’s cytotoxic properties.

## Figures and Tables

**Table 1:** Characteristics of polyelectrolytes used in the work.

Polymer	MW, kDa	Natural or Synthetic	Structure	Charge
Chitosan	5 or 7	Natural	Linear	Polycation
PEG	5	Synthetic	Linear	Nonionic
Chitosan-Glycol	72	Synthetic	Linear	Polycation
PEI	30–40	Synthetic	Linear	Polycation
PEI-PEG	13	Synthetic	Branched	Polycation
Heparin	20–50	Natural	Linear	Polycation

PEG, polyethylene glycol; PEI, polyethyleneimine.

**Table 2:** Composition of the chitosan copolymers used.

Copolymer	Modification
Chitosan–PEG (MW 5 and 5 kDa)	14 PEG chains
Chitosan–PEG (MM 7 and 5 kDa)	23 PEG chains
Chitosan–PEI (MM 7 and 30 kDa)	3 chains of PEI
Chitosan–glycol (MM 72 kDa)	NA

PEG, polyethylene glycol; PEI, polyethyleneimine.

**Table 3:** Comparative characteristics of L-asparaginases from *Rhodospirillum rubrum* and *Erwinia carotovora* [23,24].

Characteristic	RrA	EwA
MW, kDa	18	34
pI	5.1	7.6
pH optimum	9.0–9.3	8.5–9.2
Specific activity, IU/mg	56	490

**Table 4:** K_M_ and V_max_ values for native RrA and PEC–RrA-PEI within a pH range of 6.5–9.0 in a 5 mM citrate–phosphate–borate buffer.

pH	Preparation	K_M_, mM	V_max_, IU/mg	V_max_/K_M_, IU/(mg·mM)
6.5	Native RrA	6.7 ± 3.1	22 ± 4	3.3
	RrA-PEI	9.5 ± 5.0	45 ± 7	4.7
7.5	Native RrA	4.8 ± 1.6	59 ± 5	12.3
	RrA-PEI	6.5 ± 3.6	75 ± 8	11.5
9.0	Native RrA	2.9 ± 0.8	107 ± 7	36.9
	RrA-PEI	4.8 ± 1.9	139 ± 12	29.0

**Table 5:** The composition and activity of RrA conjugates. The hydrolysis activity for 20 mM L-asparagine was measured in 10 mM PBS, pH 7.5.

Preparation	Conjugate Composition	ζ-Potential	Specific Activity, IU/mg
Native RrA	NA	−8.9 ± 1.3	47 ± 4
RrA-chitosan-PEG 5_14 (5 and 5 kDa)	n(PEG)/n(chitosan) = 14 n(chitosan)/n(enzyme) = 2	NA	51 ± 3
RrA-chitosan-PEG 7_23 (7 and 5 kDa)	n(PEG)/n(chitosan) = 23 n(chitosan)/n(enzyme) = 5	−5.7 ± 1.4	62 ± 3
RrA-chitosan-glycol (72 kDa)	n(chitosan)/n(enzyme) = 3	0.2 ± 0.6	50 ± 2
RrA-chitosan-PEI (7 and 30 kDa)	n(PEI)/n(chitosan) = 3 n(chitosan)/n(enzyme) = 4	NA	55 ± 3

**Table 6:** Content of elements of secondary protein structure in L-asparaginase RrA and its conjugates with chitosan-PEG, chitosan-glycol, and chitosan-PEI determined by CD spectroscopy.

Conjugate Composition	RrA	RrA-Chitosan-PEG 7_30	RrA-Chitosan-Glycol	RrA-Chitosan-PEI
α-Helices	37 ± 2	32 ± 2	34 ± 2	33 ± 2
Parallel β-sheets	8 ± 1	9 ± 1	8 ± 1	9 ± 1
Antiparallel β-sheets	7 ± 1	9 ± 1	9 ± 1	9 ± 1
β-Turns	16 ± 1	17 ± 1	17 ± 1	17 ± 1
Unordered regions	31 ± 1	33 ± 2	33 ± 1	34 ± 2

**Table 7:** Values of the trypsinolysis rate (inhibition constant, K_in_) for the RrA conjugates.

Preparation	K_in_, min^−1^
Native RrA	0.07 ± 0.01
RrA-PEI (PEC)	0.007 ± 0.002
RrA-chitosan-glycol	0.04 ± 0.01
RrA-chitosan-PEG 7_23	0.02 ± 0.01
RrA-chitosan-PEI	0.008 ± 0.002

## Data Availability

Data is contained within the article and supplementary material.

## Supplementary Materials

The following supporting information can be downloaded at: https://www.mdpi.com/article/10.3390/ph15040406/s1, Figure S1. IR spectra: native enzyme RrA (**1**), RrA–chitosan–glycol (**2**), RrA–chitosan–PEG (7 and 5 kDa, 23 chains) (**3**). Figure S2. IR spectra of native RrA at concentrations of 2–5 mg/mL. Figure S3. Chromatogram of the native enzyme and the RrA–chitosan–PEG conjugate.
