# Supplementary material for: Improvement of Biocatalytic Properties and Cytotoxic Activity of L-Asparaginase from Rhodospirillum rubrum by Conjugation with Chitosan-Based Cationic Polyelectrolytes
Source: Pharmaceuticals (Basel). 2022 Mar 27;15(4):406. doi: 10.3390/ph15040406 (PMC9029710; doi:10.3390/ph15040406)
Supplement: Supplementary file 1 [file pharmaceuticals-15-00406-s001.zip › pharmaceuticals-1617555-supplementary.pdf]

Supplementary Materials

Improvement of Biocatalytic Properties and Cytotoxic Activity of L-asparaginase from *Rhodospirillum rubrum* by Conjugation with Chitosan-Based Cationic Polyelectrolytes

Natalia V. Dobryakova [1,2], Dmitry D. Zhdanov [2,3,*], Nikolay N. Sokolov [2], Svetlana S. Aleksandrova [2], Marina V. Pokrovskaya [2] and Elena V. Kudryashova [1,*]

1 Chemical faculty, Lomonosov Moscow State University, Leninskie Gory st. 1, Moscow 119991, Russia; natdobryak@gmail.com
2 Laboratory of medical biotechnology, Institute of Biomedical Chemistry, Pogodinskaya st. 10/8, Moscow 119121, Russia; sokolov2144@yandex.ru (N.N.S.); v-aleksandrov@yandex.ru (S.S.A.); ivan1190@yandex.ru (M.V.P.)
3 Department of Biochemistry, Peoples' Friendship University of Russia (RUDN University), Miklukho-Maklaya Street 6, Moscow 117198, Russia
* Correspondence: helena_koudriachova@hotmail.com (E.V.K.); zhdanovdd@gmail.com (D.D.Z.)

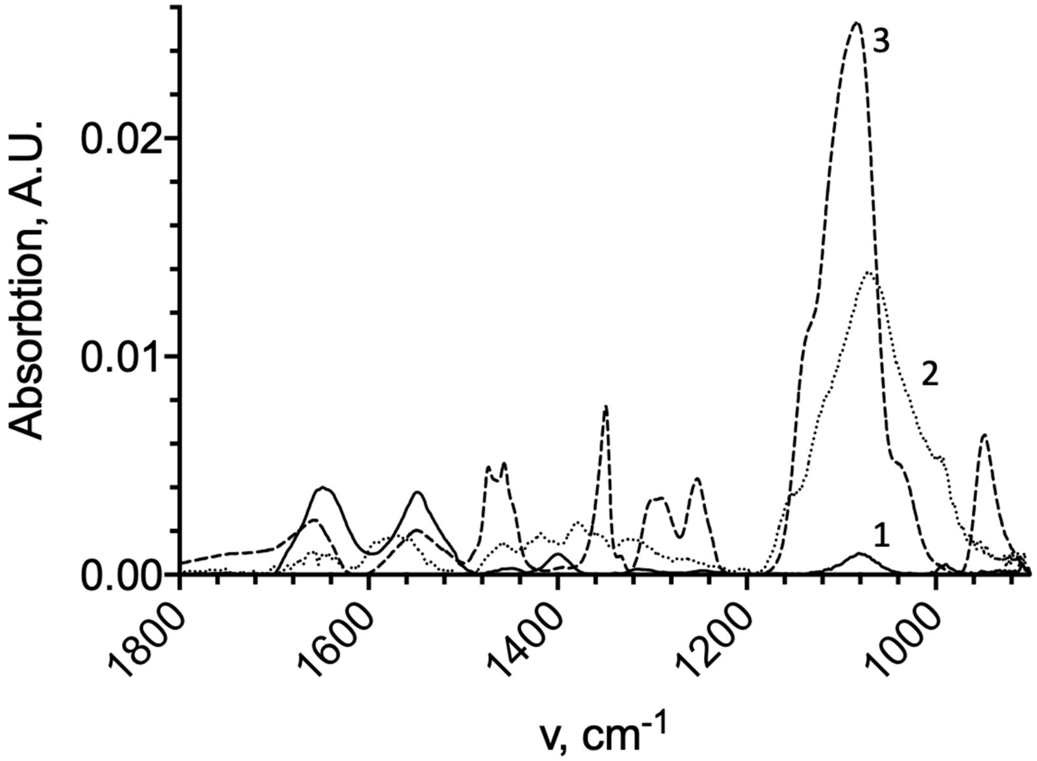

Figure S1. IR-spectra: native enzyme RrA (1), RrA-chitosan-glycol (2), RrA-chitosan-PEG (7 and 5 kDa, 23 chains) (3); sodium phosphate buffer, 10 mM, 37°C, pH 7.5

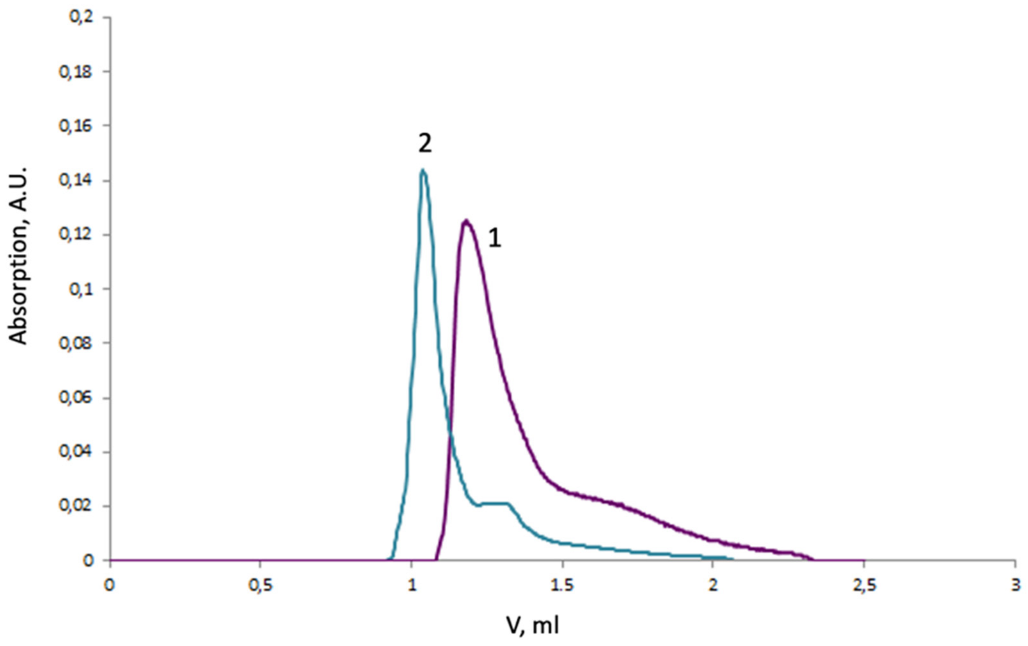

Figure S2. Chromatogram of native RrA (1) and RrA-chitosan-PEG conjugate (2). Medium: Sephadex G-200 pore size (up to 200 kDa) Eluent: Sodium phosphate buffer, pH = 7.1.

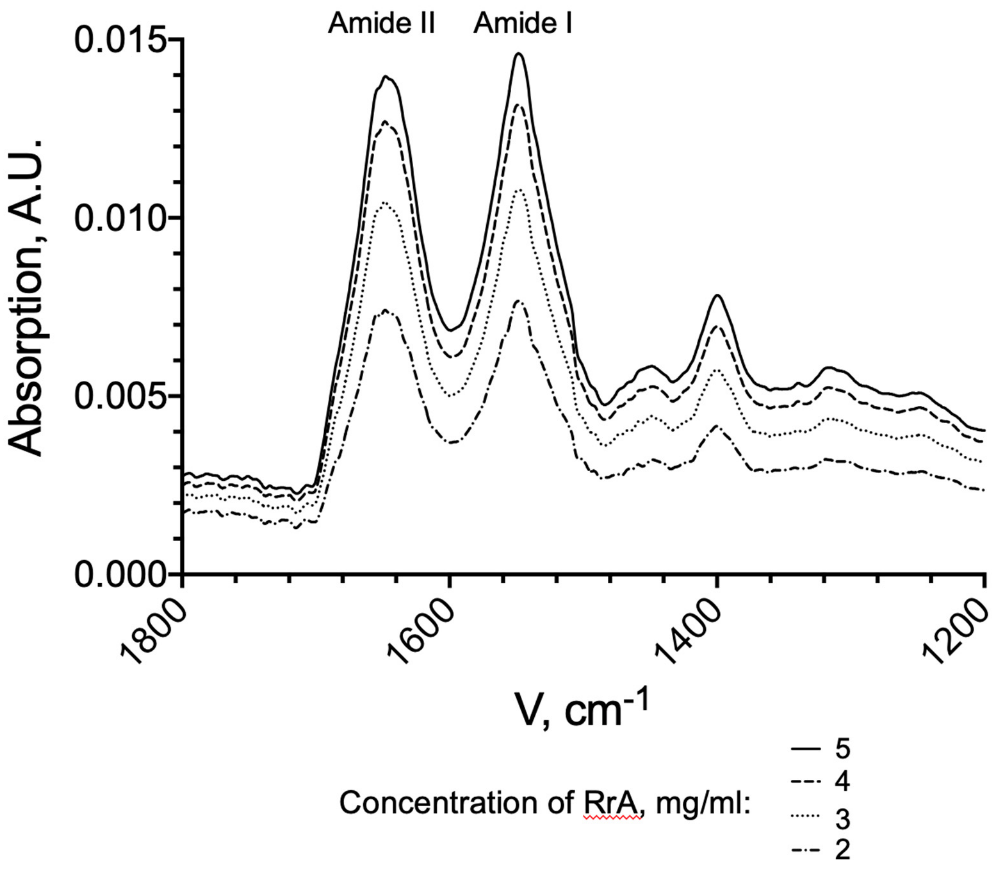

Figure S3. IR spectra of native RrA at concentrations of 2-5 mg/ml.
